# Cardiovascular medication burden in dementia disorders: a nationwide study of 19,743 dementia patients in the Swedish Dementia Registry

**DOI:** 10.1186/alzrt264

**Published:** 2014-06-16

**Authors:** Pavla Cermakova, Seyed-Mohammad Fereshtehnejad, Kristina Johnell, Bengt Winblad, Maria Eriksdotter, Dorota Religa

**Affiliations:** 1 Department of Neurobiology, Care Sciences and Society, Center for Alzheimer Research, Division for Neurogeriatrics, Karolinska Institutet, 141 57 Huddinge, Sweden; 2 International Clinical Research Center and St.Anne’s University Hospital, Pekařská 53, 656 91 Brno, Czech Republic; 3 Department of Neurobiology, Care Sciences and Society, Center for Alzheimer Research, Division of Clinical Geriatrics, Karolinska Institutet, 141 57 Huddinge, Sweden; 4 Department of Neurobiology, Care Sciences and Society, Center for Alzheimer Research, Aging Research Center, Karolinska Institutet and Stockholm University, Gävlegatan 16, 113 30 Stockholm, Sweden; 5 Department of Geriatric Medicine, Karolinska University Hospital, 141 86 Huddinge, Sweden

## Abstract

**Introduction:**

Administration of several cardiovascular drugs has an effect on dementia. We aimed to investigate whether there are differences in the use of cardiovascular medication between different dementia disorders.

**Methods:**

We obtained information about dementia patients from the Swedish Dementia Registry. Patients were diagnosed with one of these dementia disorders: Alzheimer’s disease (n = 8,139), mixed dementia (n = 5,203), vascular dementia (n = 4,982), Lewy body dementia (n = 605), frontotemporal dementia (n = 409) and Parkinson’s disease dementia (n = 405). Multivariate logistic regression analysis was performed to investigate the association between use of cardiovascular medication and dementia disorders, after adjustment for age, gender, living alone, cognitive status and total number of drugs (a proxy for overall co-morbidity).

**Results:**

Seventy percent of all the dementia patients used cardiovascular medication. Use of cardiovascular drugs is common in patients with vascular and mixed dementia. Male gender, higher age, slightly better cognitive status and living with another person was associated with use of cardiovascular medication.

**Conclusions:**

Cardiovascular medication is used extensively across dementia disorders and particularly in vascular and mixed dementia. Future research should investigate the tolerability and effectiveness of these drugs in the different dementia disorders.

## Introduction

Dementia is a devastating disease that is highly related to age. Several cardiovascular (CV) disorders have been suggested as risk factors for dementia, such as hypertension [1], hypercholesterolemia [2,3], atrial fibrillation [4] and heart failure [5]. Correct management of these conditions can slow down cognitive decline [6], reduce the risk for dementia [7] and maintain stability in patients with dementia [8]. Several CV drugs have been reported to decrease the risk of developing dementia [7,9], to improve cognition [10-12] or – on the contrary – to impair cognition [13], independent from the effect of treating CV diseases.

Treatment with antihypertensive medication was associated with a 50% reduction of dementia risk in the trial Systolic Hypertension in Europe [14]. On the other hand, The Study on Cognition and Prognosis in the Elderly did not find any positive impact of antihypertensive treatment on cognitive decline and dementia [15]. Antagonists of the renin–angiotensin–aldosterone system, such as angiotensin-converting enzyme inhibitors and angiotensin II receptor blockers, have recently gained interest in the dementia field, because the renin–angiotensin–aldosterone system is involved in several major processes such as regulation of cerebral blood flow, inflammation or memory consolidation [16-18]. Centrally active angiotensin-converting enzyme inhibitors have been shown to protect against brain injury and to slow down cognitive decline [6,19-21]. Angiotensin II receptor blockers have been associated with a significant reduction in the incidence and progression of dementia compared with other CV drugs in a prospective cohort analysis [22]. Clinical trials aimed to investigate the impact of lipid-lowering drugs on dementia have not been able to show a significant benefit on the reduction of dementia risk [23].

Alzheimer’s disease (AD) is the most prevalent dementia disorder and accounts for about two-thirds of dementia cases [24]. The second most common type is vascular dementia (VaD). Individuals with AD are often afflicted with VaD, which is then named mixed dementia (MixD) [25]. There is a lack of reliable epidemiological data on the prevalence of dementia with Lewy bodies (DLB), but this is considered the third most common type [26,27]. DLB often overlaps with Parkinson’s disease dementia (PDD) [28]. Frontotemporal dementia (FTD) accounts for about 4 to 10% of all dementia subtypes [24].

AD patients used to be considered as the healthiest group of dementia patients [29], since it has been reported that AD is associated with fewer comorbidities compared with the other dementia subtypes [30,31]. These results are not in line with more recent papers that have reported the opposite [32,33]. In a study by Imfeld and colleagues, however, CV comorbidities and exposure to CV drugs were significantly lower in AD patients, whereas the opposite result was found in VaD patients [29]. A recent study on incident AD cases showed that 34% were treated with more than five drugs [34].

The treatment of CV diseases or administration of CV drugs may be negatively influenced by several conditions associated with dementia. Autonomic dysfunction is present in all dementia disorders [35,36], particularly in DLB and PDD [37], and complicates the management of blood pressure. Furthermore, dysphagia in AD patients [38], living alone [39] and a high level of cognitive impairment [40] can cause problems with the administration of drugs.

There is an insufficient number of studies on comparisons between all different dementia disorders [41,42], probably due to the lack of large patient material. This study aims to investigate whether there are differences in the use of CV medication between different dementia disorders in a large population of dementia patients from the Swedish Dementia Registry (SveDem). To our knowledge, SveDem includes the highest number of patients with different dementia disorders worldwide.

## Methods

### Study population

We analyzed cross-sectional data from SveDem, which is a national registry for improvement of the quality of diagnostic workup, treatment and care of patients with dementia in Sweden. This registry included 28,722 patients who were newly diagnosed with dementia either at a memory clinic or in a primary care unit from 2007 to 2012. Age, gender, demographic data, body mass index, Mini Mental State Examination (MMSE) scores, diagnostic procedures, type of dementia disorder and treatment are recorded in this web-based registry.

Since 2009 SveDem has been monitored to ensure the quality of the registered data. According to a recent annual report, 76% of memory clinics are now under monitoring [43]. Ten percent of the registrations at each unit are randomly selected and checked with the data in the medical records to see whether the data correspond.

### Definitions

Dementia was diagnosed according to the International Classification of Diseases version 10 [44]. In addition, DLB was diagnosed based on the McKeith criteria [28] and the Manchester criteria were used for the diagnosis of FTD [45]. Unspecified dementia is diagnosed if the dementia etiology is unknown or if investigations aimed to differentiate the type of dementia have not been performed. Other dementia types comprise rare dementia disorders that are represented, for example, by corticobasal degeneration or alcoholic dementia. The following dementia disorders are included in SveDem: AD, VaD, MixD, LBD, FTD, PDD, unspecified dementia and other types.

In SveDem, the following classes of drugs were registered at the beginning of the diagnostic workup with the possible answers ‘yes’/‘no’/‘do not know’: CV drugs, cholinesterase inhibitors (ChEI), *N*-methyl-d-aspartate (NMDA) receptor antagonists, antidepressants, anxiolytics, antipsychotics and hypnotics/sedatives. The total number of prescribed drugs was also recorded. The latter variable was used as a proxy for overall comorbidity [46]. CV drugs comprised antihypertensives, anticoagulants, lipid-lowering drugs, antidiabetics and anti-angina medication. Living condition was reported as living alone or living with another person. This information was obtained from medical records and is therefore based on the medical history received directly from patients or indirectly; that is, from relatives or caregivers.

Only patients who were diagnosed with AD, VaD, MixD, LBD, FTD and PDD were analyzed (*n* = 21,458). Patients attributed to unspecified dementia or to other types were not included in this study due to the imprecision of these diagnostic groups. Also, patients with the answer ‘do not know’ (8%) about CV medication were excluded from this study. In total, 8,979 subjects were excluded from the whole SveDem population.

### Ethical issues

Patients and their relatives were informed orally and in writing about SveDem and could decline participation. The study was approved by the regional ethical review board in Stockholm (Drn: 2013/147-31/2). The data were anonymized before statistical analysis.

### Statistical analysis

Descriptive data are presented as the mean and standard deviation. We checked the normality of distribution using the Kolmogorov–Smirnov test. In cases of continuous variables we used the independent sample *t* test or one-way analysis of variance, and for categorical variables we used the chi-square test. Multivariate analysis was performed using binary logistic regression modeling to calculate odds ratios (ORs) with 95% confidence intervals (CIs). Two-tailed *P* < 0.05 was considered to be statistically significant in all analytical procedures. Data were analyzed using the Statistical Package for the Social Sciences software version 22 (IBM Corporation, Armonk, NY, USA).

## Results

A total of 19,743 incident dementia patients were included in this study. Their characteristics are presented in Table 1. There were 11,537 women (58%) and 8,206 men (42%). The mean age of the patients at the time of diagnosis was 78.9 ± 7.8 years and the mean MMSE score was 21 ± 5.

**Table 1:** Characteristics of the dementia patients, Swedish Dementia Registry 2007 to 2012

	**Total**	**AD**	**MixD**	**VaD**	**DLB**	**FTD**	**PDD**
	**(*n *** **= 19,743)**	**(*n *** **= 8,139; 41%)**	**(*n *** **= 5,203; 27%)**	**(*n *** **= 4,982; 25%)**	**(*n *** **= 605; 3%)**	**(*n *** **= 409; 2%)**	**(*n *** **= 405; 2%)**
**Age**							
**Mean**	78.9 ± 7.8	77.7 ± 8.1	81.0 ± 6.5	80.0 ± 7.4	76.5 ± 7.1	70.0 ± 10.0	75.2 ± 7.0
**Range**	27 to 103	27 to 99	52 to 100	33 to 103	53 to 94	39 to 96	49 to 94
**Gender, *n *** **(%)**							
**Female**	11,537	5,328	3,075	2,514	236	228	156
	(58.0%)	(65.5%)	(59.1%)	(50.5%)	(39.0%)	(55.7%)	(38.5%)
**Male**	8,206	2,811	2,128	2,468	369	181	249
	(42.0%)	(34.5%)	(40.9%)	(49.5%)	(61.0%)	(44.3%)	(61.5%)
**MMSE**							
**Mean**	21.3 ± 5.0	21.6 ± 5.0	21.0 ± 5.0	21.2 ± 4.9	21.5 ± 5.0	23.6 ± 5.0	21.0 ± 5.1
**Range**	0 to 30	0 to 30	0 to 30	0 to 30	0 to 30	3 to 30	6 to 30
**Living alone (%)**	44.3	43.0	48.7	45.7	34.7	33.0	22.7
**CV drugs (%)**	70.0	59.4	76.9	84.3	59.5	53.3	57.5
**ChEIs (%)**	49.8	73.0	53.9	7.8	74.4	6.8	54.6
**NMDA antagonists (%)**	10.5	9.8	17.0	4.7	15.0	4.9	11.6
**Number of drugs**							
**Mean**	5.0 ± 3.1	4.0 ± 2.8	5.4 ± 3.1	6.2 ± 3.2	4.8 ± 2.9	3.7 ± 2.8	6.5 ± 3.2
**Range**	0 to 24	0 to 21	0 to 24	0 to 23	0 to 17	0 to 13	0 to 19

AD, Alzheimer’s disease; ChEI, cholinesterase inhibitor; CV, cardiovascular; DLB, dementia with Lewy bodies; FTD, frontotemporal dementia; MixD, mixed dementia; MMSE, Mini Mental State Examination; NMDA, *N*-methyl-d-aspartate; PDD, Parkinson’s disease dementia; VaD, vascular dementia.

**Figure 1:**
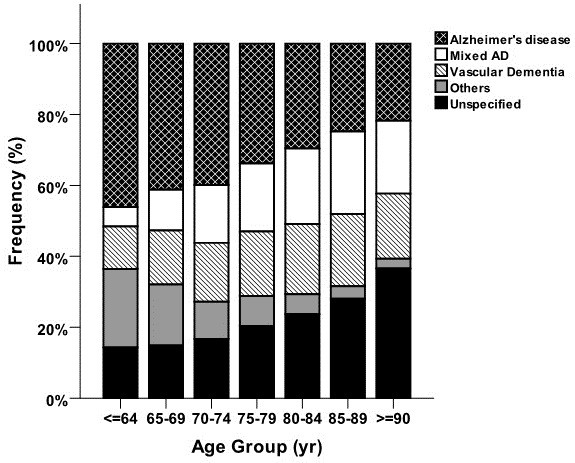
**Proportion of various types of dementia in different age groups in patients registered in the Swedish Dementia Registry during 2007 to 2012.** AD, Alzheimer’s disease; yr, year.

**Figure 2:**
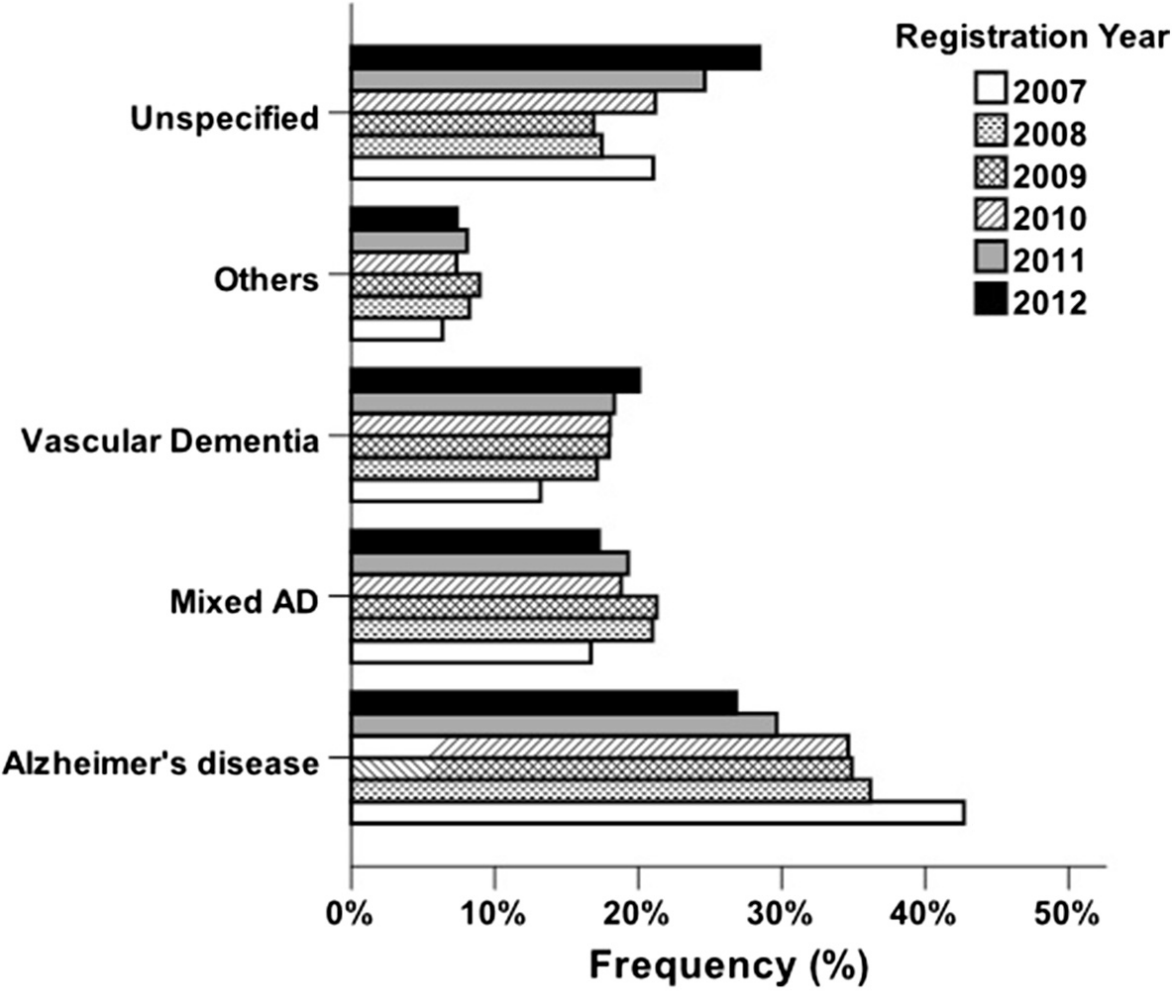
**Frequency of different types of dementia in patients registered in the Swedish Dementia Registry during 2007 to 2012 within each registration year.** AD, Alzheimer’s disease.

AD was the most common dementia disorder in the study population (41%). MixD accounted for 27% and VaD for 25% of all dementia subtypes. Other types represented a minority (DLB, 3%; FTD, 2%; and PDD, 2%). Patients diagnosed with MixD were the oldest (mean age 81.0 ± 6.5 years), while those with FTD represented the youngest group with a mean age of 70.0 ± 10.0 years. The mean MMSE score varied between 21 ± 5 in the group of MixD and 24 ± 5 in FTD patients.Figure 1 illustrates the distribution of various types of dementia in different age groups in the study population. The proportion of VaD ranged from 12 to 20.4% in different age groups and a gradual increase was observed from the youngest group (≤64 years) to the group 85 to 89 years old. The rate of patients diagnosed with AD decreased steadily from 46.1% in the ≤64 year group to 21.8% among those who were older than 90 years at the time of diagnosis. Moreover, as shown in Figure 1, the relative frequency of an unspecified type of dementia increased from 14.3% to as high as 36.6% in the oldest group. The proportion of patients diagnosed with VaD increased from 13.2% in 2007 to 20.1% in 2012 during the registration period (Figure 2). An opposite trend could be seen for the AD diagnosis, where the rate decreased from 42.7% to 26.8% from 2007 to 2012.

Seventy percent of the population used CV drugs. These drugs were least common among AD patients (59%) and most common among VaD patients (84%). ChEIs were prescribed to 50% of patients and they were used most by AD patients (73%) and least by FTD patients (7%). On the other hand, NMDA antagonists were largely used in MixD (17%) and only seldom in VaD (5%). The mean number of prescribed drugs ranged from 3.7 ± 2.8 in FTD patients to 6.5 ± 3.2 in PDD patients.

Table 2 presents the differences between patients who used CV drugs and those who did not. Patients with CV medications were older and had a slightly higher MMSE score compared with those who were not treated with CV drugs. The use of ChEIs was higher among patients who did not receive CV medication, while the opposite was found for NMDA antagonists.

**Table 2:** Characteristics of patients with and without cardiovascular drugs, Swedish Dementia Registry 2007 to 2012

	**CV medication (*n *** **= 13,847)**	**Without CV medication (*n *** **= 5,896)**	***P *** **value**
**Age**			
**Mean**	79.6 ± 7.2	77.4 ± 8.9	<0.001
**Range**	33 to 103	27 to 97	
**Gender**			
**Female (%)**	55.9	64.4	<0.001
**Male (%)**	44.1	35.6	
**MMSE**			
**Mean**	21.4 ± 4.9	21.3 ± 5.2	<0.001
**Range**	0 to 30	0 to 30	
**Living alone (%)**	43.7	45.7	0.006
**ChEIs (%)**	46.1	58.5	<0.001
**NMDA antagonists (%)**	10.6	10.3	0.028
**Number of drugs**			
**Mean**	5.8 ± 3	3,0 ± 2.5	<0.001
**Range**	0 to 24	0 to 21	
**AD (%)**	34.9	56.0	<0.001
**MixD (%)**	28.9	20.4	<0.001
**VaD (%)**	30.3	13.2	<0.001
**DLB (%)**	2.6	4.2	<0.001
**FTD (%)**	1.6	3.2	<0.001
**PDD (%)**	1.7	2.9	<0.001

AD, Alzheimer’s disease; ChEI, cholinesterase inhibitor; CV, cardiovascular; DLB, dementia with Lewy bodies; FTD, frontotemporal dementia; MixD, mixed dementia; MMSE, Mini Mental State Examination; NMDA, *N*-methyl-d-aspartate; PDD, Parkinson’s disease dementia; VaD, vascular dementia.

In the adjusted logistic regression analysis (Table 3) we found that male gender correlated with a higher probability of using CV medication when compared with women (adjusted OR = 1.45; 95% CI = 1.34 to 1.57). Living alone was associated with lower use of CV drugs (adjusted OR = 0.75, 95% CI = 0.69 to 0.81). Higher age and higher MMSE score were slightly related to the use of CV medication. There were no differences in the use of dementia medication between the patients with CV drugs and those without them. VaD showed the strongest association with use of CV drugs when compared with AD (adjusted OR = 2.14; 95% CI = 1.89 to 2.44). The probability of receiving CV medication was also significantly higher in MixD (adjusted OR = 1.57; 95% CI = 1.43 to 1.73). DLB and PDD patients were less likely to receive CV medication, while the difference between FTD and AD was not significant.

**Table 3:** Adjusted odds ratios for characteristics of patients with cardiovascular drugs compared to persons without cardiovascular medication, Swedish Dementia Registry 2007 to 2012

	**Patients with CV medication (*n *** **= 13,847)**
**Age**	1.02 (1.01 to 1.02)^*^
**Male gender**	1.45 (1.34 to 1.57)^*^
**MMSE**	1.02 (1.01 to 1.03)^*^
**Living alone**	0.75 (0.69 to 0.81)^*^
**ChEIs**	1.08 (0.98 to 1.19)
**NMDA antagonists**	0.98 (0.85 to 1.12)
**Total number of drugs**	1.53 (1.50 to 1.56)^*^
**Dementia disorder**	
**AD**	Reference
**MixD**	1.57 (1.43 to 1.73)^*^
**VaD**	2.14 (1.89 to 2.44)^*^
**DLB**	0.71 (0.58 to 0.87)^*^
**FTD**	0.91 (0.71 to 1.17)
**PDD**	0.31 (0.24 to 0.40)^*^

Age, MMSE and total number of drugs were analyzed as continuous variables. Odds ratios with 95% confidence interval adjusted for all variables in this table. AD, Alzheimer’s disease; ChEI, cholinesterase inhibitor; CV, cardiovascular; DLB, dementia with Lewy bodies; FTD, frontotemporal dementia; MixD, mixed dementia; MMSE, Mini Mental State Examination; NMDA, *N*-methyl-d-aspartate; PDD, Parkinson’s disease dementia; VaD, vascular dementia. ^*^Statistically significant at the level of *P* < 0.001.

## Discussion

### Main findings

In our study of 19,743 dementia patients, AD was the most common dementia disorder, followed by MixD and VaD. DLB, FTD and PDD were less common. In the population of patients diagnosed with a dementia disorder, the overall use of CV drugs was 70%. Another study that investigated drug use in older persons in Sweden found that 66% of them used CV medication [47]. In a population-based study performed in Finland, the proportion of older persons who used CV medicines reached 86% [48]. The prevalence of CV drugs was above 50% in all of the dementia disorders. As expected, CV drugs were the most common in patients diagnosed with VaD (84%) followed by MixD (77%). However, the overall high use of CV medication in AD, DLB, FTD and PDD implies that these patients also suffer from CV diseases to a great extent. Our finding that AD patients seemed to have comparably less CV comorbidities may be biased by the fact that dementia patients with CV comorbidities are more likely to be diagnosed with VaD or MixD than with AD. The relationship between CV diseases and AD is complex and may be influenced not only by CV medication, but also by anti-dementia medication such as ChEI. We have recently shown, using data from the SveDem registry, that treatment with ChEI in AD patients was associated with a reduced risk for myocardial infarction and death [49].

The burden of CV diseases in PDD is clinically relevant, especially in relation to treatment of Parkinson’s disease. Some dopamine agonists are associated with a worse CV profile [50] and increased risk for heart valvular fibrosis [51,52]. We observed a very high total number of drugs in PDD patients (6.5 ± 3.2). This is consistent with previous research indicating that Parkinson’s disease patients have a higher number of comorbidities compared with the general population [53]. However, PDD patients were significantly less likely to receive treatment with CV drugs when compared with AD patients. The DLB patients in our study population had a lower level of overall comorbidity. There is a lack of studies about the significance of CV diseases in DLB patients [54], but history of stroke has been reported to occur more often in DLB patients than in controls [55]. The relatively lower use of CV drugs in PDD and DLB patients compared with AD may be attributed to the fear of their side effects and difficulties in the management of blood pressure due to autonomic dysfunction [37].

Patients diagnosed with FTD represent the youngest group of dementia patients. They also seemed to have the least CV comorbidities, but after adjustment for age and other confounders they had a similar probability of receiving CV medication as AD patients (adjusted OR = 0.91; 95% CI = 0.71 to 1.17). This confirms an already known relationship between advanced age and a higher amount of comorbidities [56].

One study has suggested that men suffering from dementia have a higher level of comorbidities [57]. Our investigation confirms this finding as male gender was associated with the use of CV drugs. However, this seems to be specific for CV medication, because older women use more medicines in general than older men [58-60]. Persons using CV drugs were found to have a lower probability of living alone, which could indicate that living alone would be associated with better CV health. However, people who live alone may not seek medical treatment or may have inadequate compliance with drug treatment. Indeed, it has been shown that living with another person is associated with increased adherence to medication [61]. There are gender differences regarding living conditions in older people. Older men are more likely to live at home while older women are more often institutionalized [62]. This could be explained by differences in social and marital conditions between older men and older women [63,64] as well as by the fact that older women experience more disabilities compared with men [57]. In another investigation that compared medication in institutionalized and community-dwelling older people, institutionalization was found to be negatively associated with the use of many CV drugs [65].

### Strengths and limitations

The findings of this study are strengthened by a large sample of patients from several parts of Sweden and different dementia subtypes. Compared with drug registry-based studies that use medicines prescribed to patients, our study may reflect the reality by having information about the drug treatment obtained directly from patients or their relatives. This study is cross-sectional; thus we cannot conclude any causal relationships. However, patients in SveDem are followed-up, which opens up possibilities for longitudinal studies in the future.

We did not include persons who were attributed to unspecified or other types of dementia (*n* = 7,264; mean age 80.6 ± 8.1; mean MMSE 20.5 ± 5.2). Furthermore, in SveDem there is a possibility to respond with the answer ‘do not know’, which led to missing values for our data on CV medication and the necessity to exclude 8% of patients from the study. The persons excluded due to missing values differed from the study population in their mean age (77.6 ± 9.1 vs. 78.9 ± 7.8; *P* < 0.001) and MMSE score (21.0 ± 5.8 vs. 21.3 ± 5.0; *P* < 0.001), which could have introduced selection bias.

Patients at 58 memory clinics from all Sweden hospitals and from local to university hospitals were included. In the last year, the number of patients diagnosed in primary care units has significantly increased in SveDem and even more primary care units will be affiliated in the future. The reliability of the data in SveDem has been validated, especially in memory clinics. There is still a lower number of primary care units where the quality of registered data is monitored [43]. The data registered in memory clinics in a random sample of patients were in good agreement with medical records in a reliability test [66]. The validity of the diagnosis of dementia disorders has not been examined.

Considering the coverage of SveDem, our study could not provide incidence rates of dementia for the entire Swedish population. The incidence rate of dementia in Sweden is estimated to be about 24,000 new cases annually [67] and, considering the fact that nearly 8,000 newly diagnosed dementia patients are registered each year in SveDem, an overall coverage rate of 33% could be assumed. A recent study showed a prevalence rate of 17 to 18% in the Swedish population aged >75 years using data from two cohorts [68]. The study also concluded that the incidence of dementia might have decreased over the two recent decades. With regard to the frequency of various types of dementia within different age strata, our findings showed that the proportions of dementia patients diagnosed with VaD and unspecified dementia are increasing by age. Another previous report from Sweden claimed that VaD contributed to one-quarter of all dementias [69], which was found to be almost one-fifth or less in our study. However, in this previous survey there was no MixD included, which could have led to a higher incidence for VaD. In line with this previous report [69], we also showed that the proportion of unspecified dementia was increasing among the older age groups. Furthermore, a steady increase was observed in the proportion of VaD in our study population over time during the registration period. Although this pattern could be seen in data from both primary care units and memory clinics, the simultaneous increase in the number of primary care units and older age of registered patients might have contributed to this increase in the proportion of VaD over time.

## Conclusion

CV medication is used extensively across dementia disorders, and particularly in VaD and MixD. Future research should investigate the tolerability and effectiveness of these drugs in the different dementia disorders.

## Abbreviations

AD: Alzheimer´s disease; ChEI: Cholinesterase inhibitor; CI: Confidence interval; CV: Cardiovascular; DLB: Dementia with Lewy bodies; FTD: Frontotemporal dementia; MixD: Mixed dementia; MMSE: Mini mental state examination; NMDA: *N*-methyl-d-aspartate; OR: Odds ratio; PDD: Parkinson’s disease dementia; SveDem: Swedish dementia registry; VaD: Vascular dementia.

## Competing interests

The authors declare that they have no competing interests.

## Authors’ contributions

PČ carried out the analysis, interpreted the results and wrote the manuscript. S-MF contributed to the statistical analysis and interpretation of the data. KJ participated in the design of the manuscript and contributed substantially to its content. BW participated in the coordination of the study and revised the manuscript critically for important intellectual content. ME conceived of the study, contributed to its design and drafting the manuscript. DR participated in the study design and its coordination. All authors approved the final version of the manuscript.
