# Erratum to: Cardiovascular medication burden in dementia disorders: a nationwide study of 19, 743 dementia patients in the Swedish Dementia Registry

**DOI:** 10.1186/s13195-014-0063-4

**Published:** 2014-09-20

**Authors:** Pavla Cermakova, Seyed-Mohammad Fereshtehnejad, Kristina Johnell, Bengt Winblad, Maria Eriksdotter, Dorota Religa

**Affiliations:** 1 Department of Neurobiology, Care Sciences and Society, Center for Alzheimer Research, Division of Neurogeriatrics, Karolinska Institutet, Huddinge, 141 57, Sweden; 2 International Clinical Research Center and St.Anne’s University Hospital, Pekařská 53, Brno, 656 91, Czech Republic; 3 Department of Neurobiology, Care Sciences and Society, Center for Alzheimer Research, Division of Clinical Geriatrics, Karolinska Institutet, Stockholm, 141 57, Huddinge, Sweden; 4 Department of Neurobiology, Care Sciences and Society, Center for Alzheimer Research, Aging Research Center, Karolinska Institutet and Stockholm University, Gävlegatan 16, Stockholm, 113 30, Sweden; 5 Department of Geriatric Medicine, Karolinska University Hospital, Stockholm, 141 86, Huddinge, Sweden

## Abstract

No abstract.

## Erratum

Due to an oversight, the Acknowledgments section of the above article [1] incorrectly omitted support for DR by the Swedish Research Council (grant 2012-2291), the Swedish Society of Medicine and Alzheimerfonden. The correct statement should read:
